# Temporal and spatial analysis of low birth weight cases in Sergipe^*^

**DOI:** 10.1590/1518-8345.8113.4870

**Published:** 2026-07-24

**Authors:** Vanessa dos Santos Viana, Juliana Schaia Rocha Orsi, Regiane Cristina do Amaral

**Affiliations:** 1 Universidade Federal de Sergipe, Aracaju, SE, Brazil.; 2 Scholarship holder at the Conselho Nacional de Desenvolvimento Científico e Tecnológico (CNPq), Brazil.; 3 Pontifícia Universidade Católica do Paraná, Paraná, PR, Brazil.; 4 Universidade Federal de Sergipe, Departamento de Odontologia, Aracaju, SE, Brazil.

**Keywords:** Premature Newborn, Prenatal Care, Pregnancy, Spatial Analysis, Premature Birth, Temporal Distribution.

## Abstract

**Objective:**

to conduct a temporal and spatial time-series analysis of low birth weight cases in the Sergipe state from 2013 to 2023, identifying regions with the highest rates.

**Method:**

data from live births in the state, provided by the State Health Department, were analyzed. Kernel density maps were generated by municipality, and time-series analyses were conducted by health region.

**Results:**

between 2013 and 2023, the state recorded 370,329 births, of which 30,999 newborns weighed less than 2,500 grams (8.4%). According to the Kernel density map, the municipalities of Nossa Senhora do Socorro followed by Propriá presented the highest LBW rates. Time-series analysis showed a decreasing trend in the Nossa Senhora da Glória and Nossa Senhora do Socorro regionals.

**Conclusion:**

LBW cases were spatially clustered, and most health regions exhibited stationary trends.

## Highlights

**(1)** Identification of health regions with the highest density of LBW cases. **(2)** Most state regions showed a stationary trend in LBW rates. **(3)** Sergipe state presents high social vulnerability. **(4)** Sergipe state showed an increase in the percentage of LBW cases (2015, 2018, 2023).

## Introduction

Birth weight is a newborn’s weight at first weighing, performed within 1 hour of delivery. According to the World Health Organization (WHO)^1^, the percentage of low birth weight (LBW) infants is a significant public health indicator, as it reflects multiple factors associated with the gestational period, including maternal malnutrition, clinical and obstetric conditions, quality of prenatal care, income level, maternal age, multiple pregnancies, and nutritional status.

In 2020, the United Nations Children’s Fund (UNICEF)^2^ estimated that 19.8 million newborns-about 14.7% of all live births worldwide-presented LBW. These infants have a higher risk of dying within the first month of life, while survivors face lifelong consequences such as inadequate growth, lower IQ, and increased risk of chronic diseases in adulthood, including obesity and diabetes^3^ ^-^ ^4^. Although global UNICEF data indicate a slight decline in LBW percentages, regional disparities persist. North America and Europe maintained stable rates around 7.4% in 2000, 2010, and 2020, whereas South America saw a modest increase (8.5%, 8.6%, and 8.8%, respectively).

In Brazil^5^ ^-^ ^6^, LBW prevalence in 2023 was approximately 9.3%. The Southeast registered the highest rate (10.1%), followed by the Midwest (9.5%), South (9.3%), Northeast (8.9%), and North (8.7%). Within the Northeast, Bahia (9.65%), Ceará (9.1%), Piauí and Rio Grande do Norte (9.04%), and Sergipe (8.9%) stood out. Historical analysis shows a slight increase in LBW between 2015 and 2023: percentages in the Northeast rose from 7.9% (2015 and 2018) to 8.9% (2023), whereas Sergipe reported 8.2%, 8.3%, and 8.9% in the same periods^7^.

WHO established the goal of reducing by 30% the number of newborns weighing less than 2,500 g by 2025, corresponding to a 3% annual reduction between 2012 and 2025, lowering cases from 20 million to approximately 14 million^8^. In Brazil, one strategy to reduce LBW is beginning prenatal care in the first trimester and including at least six visits: one in the first trimester, two in the second, and three in the third^9^. The Ministry of Health also implemented programs such as *Rede Cegonha* ^10^, within the Unified Health System (SUS), to improve access, quality, and continuity of maternal and child care. However, disparities remain across regions and socioeconomic groups.

Social vulnerability is another determinant of LBW risk. According to the Public Leadership Center (CLP)^11^, when evaluating health and vulnerability indicators, Sergipe ranks 22nd among the 27 Brazilian states. Moreover, the report “Multiple Dimensions of Poverty in Childhood and Adolescence in Brazil”^12^ revealed that 76.6% of children and adolescents in Sergipe experience at least one dimension of poverty, and 22.68% live in extreme poverty.

Given the epidemiological and social relevance of the problem, this study analyzed the temporal trend and spatial distribution of LBW cases in Sergipe from 2013 to 2023, identifying regions with the highest rates.

## Methods

### Location

Spanning 182,163 km² and located in the Northeast region, Sergipe is the smallest state in Brazil. It has an estimated population (2024) of 628,846 inhabitants and a Human Development Index (HDI) of 0.77. In 2022, infant mortality was 16.81 deaths per 1,000 live births^13^.

The state’s Maternal and Child Health Network consists of nine maternity hospitals (five public, four philanthropic)-three are located in the state capital, Aracaju, and six in regional hub municipalities (Estância, Itabaiana, Lagarto, Nossa Senhora da Glória, Nossa Senhora do Socorro, and Propriá)^14^.

### Sample definition

A retrospective epidemiological study was conducted using secondary data obtained from the Sergipe State Health Department on live births between 2013 and 2023. The study was approved by a Research Ethics Committee (CAAE: 77636824.5.0000.5546; decision no. 6.684.748).

Data were obtained from the Live Birth Information System (SINASC), including: place of birth, maternal age, race/ethnicity, marital status, schooling level, gestational age, parity, delivery type, prenatal consultations, date of birth, gender of the newborn, birth weight, and gestational week.

Inclusion criteria consisted of records with birth weight information. Variables included: municipality of birth, sociodemographic factors (age, marital status, schooling level, ethnicity, gender of the newborn, congenital anomalies), gestational age, parity, type of delivery, and number of prenatal consultations.

SINASC registered a total of 370,413 births from 2013 to 2023, of which 370,329 had the birth weight recorded. Of these, 30,999 infants were born weighing <2,500 g, representing 8.4% of all births.

### Data processing and analysis

LBW rate was calculated as the number of infants <2,500 g divided by the total number of births. Rates were computed for each municipality. Infants born before 37 weeks were classified as preterm. Missing or incomplete data were excluded.

Kernel density estimation maps (point density over geographic space) were produced using a 20,000 m radius, 80-pixel resolution, and a continuous interval mode on SIRGAS 2000/UTM zone 24S. Analyses were performed on QGIS 3.22.5.

Time-series trend analysis used annual average percent change (AAPC) at the 95% significance level considering seven health regions (Aracaju, Estância, Itabaiana, Lagarto, Nossa Senhora da Glória, Nossa Senhora do Socorro, and Propriá). Prais-Winsten regression was applied to correct first-order autocorrelation. The dependent variable was log-transformed case counts; the independent variable was year, as in Antunes and Waldman^15^. Analyses were performed on Stata 14.

Health regions consist of neighboring municipalities sharing cultural, economic, and social identities and transport infrastructure. Regional structuring is guided by the Tripartite Intermanagement Committee (CIT) Resolution 1/2011 and Presidential Decree 7.508/2011^16^.

## Results

Among LBW cases, mean maternal age was 26 years (±7.4), ranging from 10 to 53 years. Of the newborns, 16,365 were female, 14,562 were male, and 72 had their gender listed as unknown. Table 1 shows that most mothers were of mixed race, single, had 8-11 years of schooling, and delivered at 32-36 weeks.

**Figure 1:**
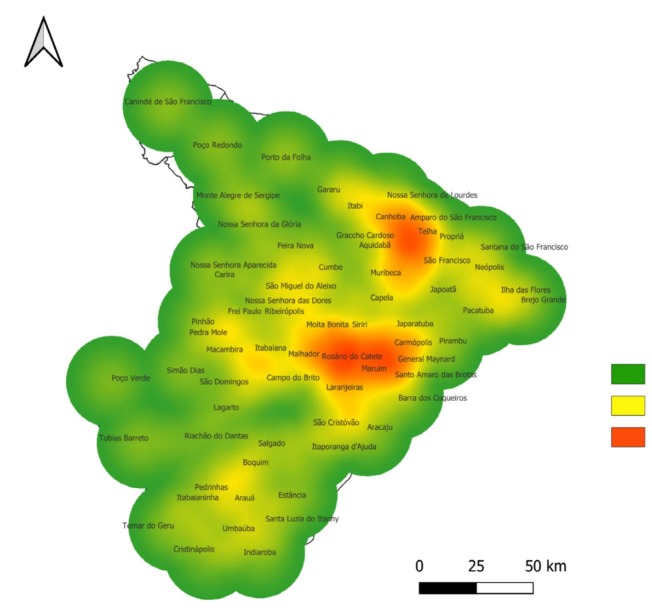
Kernel density map of low birth weight rate by municipality in the State of Sergipe, from 2013 to 2023. Aracaju, SE, Brazil, 2024

Kernel density analysis identified the municipalities of Nossa Senhora do Socorro region followed by the Propriá region as the highest LBW concentration. The red and yellow colors on the map represent values above state averages (Figure 1).

Time-series analysis revealed decreasing LBW trends in the Nossa Senhora da Glória and Nossa Senhora do Socorro regions (Table 2).

**Table 1:** Sociodemographic variables of the total* number of low birth weight (LBW) newborns in the State of Sergipe, 2013-2023. Aracaju, SE, Brazil, 2024

Maternal age	N*	%
10 to 17 years	3,856	12.4
18 to 35 years	23,025	74.3
More than 35 years	4,118	13.3
**Race**		
White	2,923	9.58
Black	1,600	5.24
Asian	265	0.87
Mixed race	25,663	84.12
Indigenous	55	0.18
**Marital status**		
Single	18,102	58.61
Married	5,992	19.40
Widowed	68	0.22
Separated/Divorced	289	0.94
Stable Union	6,434	20.83
**Maternal schooling**		
None	280	0.9
1 to 3 years	1,574	5.1
4 to 7 years	7,888	25.5
8 to 11 years	16,633	53.8
12 or more	4,518	14.6
**Type of pregnancy**		
Single	26,568	74.7
Double	8,550	24.0
Triple or more	441	1.2
**Prenatal consultations**		
None	806	0.8
1 to 3	10,672	10.8
4 to 6	36,042	36.6
7 or more	50,984	51.8
**Gestational weeks**		
Less than 22	342	1.11
22 to 27	1,647	5.37
28 to 31	2,659	8.67
32 to 36	15,281	49.81
37 to 41	10,604	34.56
42 or more	146	0.48

*Total of 30,999 LBW newborns. Lower totals indicate missing data

**Table 2:** Time series analysis of LBW cases by health regions of Sergipe, from 2013 to 2023. Aracaju, SE, Brazil, 2024

Health Region	AAPC*	CI^†^ (min^‡^-max^§^)	Trend
Aracaju	-0.36	-0.02 - 0.01	stationary
Estância	-2.24	-0.08 - 0.04	stationary
Itabaiana	-0.90	-0.02 - 0.01	stationary
Lagarto	-1.07	-0.05 - 0.03	stationary
Nossa Senhora da Glória	-3.04	-0.05 - -0.01	decreasing
Nossa Senhora do Socorro	-6.82	-0.10 - -0.03	decreasing
Propriá	-3.95	-0.09 - 0.01	stationary

*AAPC = Annual average percent change; ^†^CI = Confidence interval; ^‡^min = Minimum value; ^§^max = Maximum value

## Discussion

Low birth weight (LBW) constitutes a major public health issue with significant consequences for infant survival and long-term health. Understanding regional patterns is key to informing targeted interventions^17^.

LBW is associated with multiple factors, including prematurity, inadequate prenatal care, maternal age extremes, marital status, low schooling level, parity, history of miscarriages, prior LBW infants, female sex, and social vulnerability^18^ ^-^ ^20^.

In the present study, most LBW newborns were preterm, female, and born to single mothers. Other studies found associations between term LBW and male sex, multiparity, fewer prenatal visits, and adolescent parents, with most mothers having primary education and being single^21^ ^-^ ^22^. Race has also been shown to correlate with LBW, especially among socioeconomically vulnerable Black and mixed-race women^22^, supporting the present findings.

Other authors^23^ found associations between LBW, maternal age, anemia, and socioeconomic deprivation. The maternal age extremes (10 and 53 years) identified here underscore the influence of such factors.

Kernel density maps are widely used to visualize the spatial distribution of events, helping identify hotspots and guide health planning^24^ ^-^ ^25^. In this study, the municipalities with the highest LBW density-Rosário do Catete, Telha, and Divina Pastora-are small cities with low HDI values (0.60-0.63)^13^, consistent with the literature linking low HDI to poorer health outcomes^24^ ^-^ ^26^.

In 2023, Brazil recorded an infant mortality rate of 12.6/1,000 live births; in Sergipe, this rate was 18.48/1,000 live births. All highlighted municipalities exceeded national averages, reflecting heightened vulnerability.

LBW prevalence in Sergipe (8.4%) aligns with Latin America (8.7%) and with Brazil overall (8%)^27^. Despite a downward trend in infant mortality, it remains elevated (13.3/1,000 births)^5^, reinforcing the need for strengthened care.

Most health regions showed stationary LBW trends which, indicating no significant improvement, signal a need for stronger interventions. Even LBW hotspot areas showed stationary or decreasing trends, highlighting municipal-level disparities.

Nonetheless, some state indicators meet targets, such as timely prenatal care initiation (52% before 12 weeks) and high primary care coverage (118% in 2025).

Important study limitations include possible inconsistencies in SINASC reporting and the ecological study design, which prevents evaluating individual-level maternal health factors.

## Conclusion

This study identified stationary trends in most health regions and spatial clusters of LBW in certain municipalities, highlighting areas requiring greater health actions and investigation.

## Data Availability

All data generated or analysed during this study are included in this published article.
