# Supplementary material for: Temporal and spatial analysis of low birth weight cases in Sergipe
Source: Rev Lat Am Enfermagem. 2026 Jul 24;34:e4870. doi: 10.1590/1518-8345.8113.4870 (PMC13405587; doi:10.1590/1518-8345.8113.4870)
Supplement: Translation

# Translation: Análise temporal e espacial das taxas de baixo peso ao nascer em Sergipe^*^

**DOI:** 10.1590/1518-8345.8113.4871

**Authors:** Vanessa dos Santos Viana, Juliana Schaia Rocha Orsi, Regiane Cristina do Amaral

**Affiliations:** 1 Universidade Federal de Sergipe, Aracaju, SE, Brasil.; 2 Scholarship holder at the Conselho Nacional de Desenvolvimento Científico e Tecnológico (CNPq), Brasil.; 3 Pontifícia Universidade Católica do Paraná, Paraná, PR, Brasil.; 4 Universidade Federal de Sergipe, Departamento de Odontologia, Aracaju, SE, Brasil.

**Keywords:** Recém-Nascido Prematuro, Cuidado Pré-Natal, Gravidez, Análise Espacial, Nascimento Prematuro, Distribuição Temporal.

## Abstract

**Objetivo:**

realizar uma análise de séries temporais e espaciais dos casos de baixo peso ao nascer no estado de Sergipe no período de 2013 a 2023, identificando as regiões com maiores taxas.

**Método:**

foram utilizados dados referentes aos nascidos vivos neste estado, fornecidos pela Secretaria Estadual de Saúde. Realizaram-se análises de mapa de Kernel por município do estado e de séries temporais por regiões de saúde.

**Resultados:**

no período, foram observados 370.329 nascimentos, dos quais 30.999 nasceram com peso menor que 2.500 gramas, o que representa 8,4%. De acordo com o mapa de Kernel, os municípios com maior destaque na taxa de BPN foram os da regional de Nossa Senhora do Socorro, seguida pela regional de Propriá. Ao se realizar a análise de séries temporais, verifica-se uma tendência decrescente para as regiões de Nossa Senhora da Glória e de Nossa Senhora do Socorro.

**Conclusão:**

os casos mostram-se em densidade localizada e com tendência estacionária na maioria das regionais de saúde analisadas.

## Destaques

**(1)** Foram identificadas regiões de saúde com maior densidade de casos de BPN. **(2)** A maioria das regionais do Sergipe apresenta-se com tendência estacionária das taxas de BPN. **(3)** O estado de Sergipe apresenta-se com grande vulnerabilidade social. **(4)** O Sergipe apresentou aumento do percentual dos casos de BPN (2015, 2018, 2023).

## Introdução

O peso ao nascer corresponde à primeira pesagem do recém-nascido realizada até uma hora após o parto. De acordo com a Organização Mundial da Saúde (OMS)^1^, a proporção de bebês com baixo peso ao nascer (BPN) é um indicador importante de saúde pública, pois reflete múltiplos fatores associados ao período gestacional, como desnutrição materna, condições clínicas e obstétricas, qualidade da assistência pré-natal, nível de renda, idade materna, gestações múltiplas e estado nutricional.

Em 2020, o Fundo das Nações Unidas para a Infância (UNICEF)^2^ estimou que 19,8 milhões de recém-nascidos - cerca de 14,7% de todos os nascidos vivos no mundo - apresentaram BPN. Esses bebês apresentam maior risco de morrer no primeiro mês de vida e, entre os sobreviventes, são observadas consequências ao longo do ciclo vital, como maior probabilidade de crescimento inadequado, menor quociente intelectual e maior risco de doenças crônicas na idade adulta, incluindo obesidade e diabetes^3^ ^-^ ^4^. Embora os dados globais da UNICEF indiquem uma tendência de redução do percentual de BPN, persistem diferenças regionais significativas. A América do Norte e a Europa mantiveram índices estáveis em torno de 7,4% nos anos de 2000, 2010 e 2020, enquanto a América do Sul apresentou um discreto aumento (8,5%, 8,6% e 8,8%, respectivamente).

No Brasil^5^ ^-^ ^6^, a prevalência de BPN em 2023 foi de aproximadamente 9,3%. A região Sudeste apresentou a maior taxa (10,1%), seguida pelo Centro-Oeste (9,5%), Sul (9,3%), Nordeste (8,9%) e Norte (8,7%). Na região Nordeste, destacaram-se Bahia (9,65%), Ceará (9,1%), Piauí e Rio Grande do Norte (9,04%) e Sergipe (8,9%). A análise histórica revela um pequeno aumento no percentual de BPN entre 2015 e 2023: o Nordeste passou de 7,9% (2015 e 2018) para 8,9% (2023), enquanto Sergipe registrou 8,2%, 8,3% e 8,9% nesses mesmos períodos^7^.

A OMS estabeleceu como meta uma redução de 30% no número de bebês com peso inferior a 2.500 gramas até 2025, o que equivale a uma queda relativa de 3% ao ano entre 2012 e 2025, passando de 20 para cerca de 14 milhões de recém-nascidos com BPN^8^. Como medida para a diminuição dos BPN, no Brasil, recomenda-se que o pré-natal tenha início no primeiro trimestre e inclua, no mínimo, seis consultas: uma no primeiro trimestre, duas no segundo e três no terceiro^9^. Além disso, o Ministério da Saúde implementou programas, como a Rede Cegonha^10^, no âmbito do Sistema Único de Saúde (SUS), visando melhorar o acesso, a qualidade e a continuidade do cuidado materno-infantil. Contudo, apesar desses avanços, ainda se observam disparidades regionais e socioeconômicas na prevalência de BPN.

A vulnerabilidade social representa outro importante determinante do BPN. Segundo o Centro de Liderança Pública (CLP)^11^, ao se avaliar indicadores de saúde e de vulnerabilidade, Sergipe ocupa a 22ª posição entre os 27 estados brasileiros. Além disso, o relatório *As Múltiplas Dimensões da Pobreza na Infância e Adolescência no Brasil* ^12^ revelou que 76,6% das crianças e adolescentes sergipanos vivenciam pelo menos uma dimensão da pobreza, e 22,68% encontram-se em pobreza extrema.

Assim, diante da relevância epidemiológica e social do problema, este estudo teve como objetivo analisar a tendência temporal e a distribuição espacial dos casos de BPN no estado de Sergipe de 2013 a 2023, identificando as regiões com maiores taxas.

## Método

### Local 

Sergipe é o menor estado da Federação, com 182.163 km², situado no Nordeste brasileiro, e tem uma população estimada (2024) de 628.846 pessoas e Índice de Desenvolvimento Humano (IDH) igual a 0,77. A mortalidade infantil no estado em 2022 foi de 16,81 óbitos por mil nascidos vivos^13^.

A Rede Materno-Infantil de Sergipe é composta por nove maternidades (cinco públicas e quatro filantrópicas). Três maternidades estão situadas na capital (Aracaju) e seis estão localizadas nos municípios-sede de cada região de saúde (Estância, Itabaiana, Lagarto, Nossa Senhora da Glória, Nossa Senhora do Socorro e Propriá)^14^. 

### Definição da amostra

Trata-se de um estudo epidemiológico, retrospectivo, com uso de dados secundários da Secretaria Estadual de Sergipe, sobre o número de nascidos vivos no período de 2013 a 2023. O estudo foi aprovado pelo Comitê de Ética em Pesquisa (CEP), sob o número CAAE: 77636824.5.0000.5546, parecer nº 6.684.748.

Os dados foram fornecidos pela Secretaria Estadual de Saúde de Sergipe, banco Sistema de Informação sobre Nascidos Vivos (SINASC), com dados informando: local de nascimento, idade da mãe, raça da mãe, estado civil da mãe, escolaridade da mãe, duração da gestação, tipo da gestação, tipo de parto, consultas, data de nascimento, sexo da criança, peso da criança, semana gestacional.

Como critério de elegibilidade, foram incluídos na amostra os dados em que o peso do bebê foi informado. Os dados incluídos foram: município de nascimento, dados sociais (idade, estado civil, escolaridade, etnia, sexo do bebê, presença de anomalia), tempo gestacional, número de gestações, tipo de parto (cesárea ou vaginal) e número de consultas pré-natais.

Foram registrados, no período, 370.413 nascimentos; contudo, 370.329 informaram o peso do bebê ao nascer; os demais constaram como não preenchidos. Do total de nascimentos, 30.999 nasceram com peso menor que 2.500 gramas, ou seja, BPN, representando 8,4% do total de nascidos. 

### Tratamento e análise dos dados

Foi calculada a taxa de BPN, definida como: total de crianças com peso menor que 2.500 gramas (OMS)/total de nascimentos. Essa taxa foi calculada por município do estado de Sergipe. Em relação ao período gestacional, observado para analisar a associação com BPN, o bebê é considerado prematuro quando nasce com menos de 37 semanas. Para os casos considerados ignorados ou incompletos, os dados foram excluídos.

Foram realizados mapas de estimativa de densidade de Kernel (densidade de pontos em uma área geográfica), utilizando raio de 20 mil metros e tamanho de pixel de 80, no modo de intervalo contínuo, com coordenadas SIRGAS 2000/UTM, zona 24S. Para tal, foi utilizado o programa *Quantum Geographic Information System* (QGIS) 3.22.5. Utilizou-se essa estimativa para identificar quais os municípios com maior taxa de BPN, uma vez que, a partir dela, é possível visualizar e interpretar os dados geográficos de forma mais precisa e eficiente, auxiliando na tomada de decisões e no planejamento de ações.

Foram ainda realizadas análises de tendência temporal, com variação de percentual anual (VPA), com nível de significância de 95%, do número de casos de BPN, pelas sete regionais de saúde de Sergipe (Aracaju, Estância, Itabaiana, Lagarto, Nossa Senhora da Glória, Nossa Senhora do Socorro e Propriá). Para esta análise, foi aplicada a regressão de Prais-Winsten, que prevê a correção da autocorrelação de primeira ordem. A variável dependente foi o logaritmo dos casos, e as variáveis independentes foram os anos da série histórica, utilizando a metodologia descrita por Antunes e Waldman^15^. A análise foi realizada no programa Stata, versão 14.

As regiões de saúde são formadas por municípios fronteiriços que compartilham identidades culturais, econômicas e sociais, redes de comunicação e infraestrutura de transportes. A finalidade dessas regiões é integrar a organização, o planejamento e a execução de ações e serviços de saúde. As diretrizes de funcionamento dessas regiões foram estabelecidas pela Comissão Intergestores Tripartite (CIT), por meio da Resolução n. 1 de 29 de setembro de 2011, e em conformidade com o Decreto n. 7.508 de 28 de junho de 2011 - que organiza o SUS, o planejamento da saúde, a assistência à saúde e a articulação entre governo federal, estados e municípios^16^. 

## Resultados

Entre os BPN, a idade média materna foi de 26 anos (±7,4 anos), sendo a mãe mais jovem, com 10 anos, e a mais velha, com 53 anos. Sobre o sexo do bebê, 16.365 eram do sexo feminino; 14.562, do sexo masculino; e 72 estavam como sexo ignorado. Verifica-se, pela Tabela 1, que a maioria das mães era de raça parda, solteira, com 8 a 11 anos de estudo e 32 a 36 semanas gestacionais.

**Tabela 1:** Variáveis sociodemográficas do total* de baixo peso ao nascer (BPN) no estado de Sergipe, 2013-2023. Aracaju, SE, Brasil, 2024

Idade materna	N*	%
10 a 17 anos	3.856	12,4
18 a 35 anos	23.025	74,3
Mais que 35 anos	4.118	13,3
**Raça**		
Branca	2.923	9,58
Preta	1.600	5,24
Amarela	265	0,87
Parda	25.663	84,12
Indígena	55	0,18
**Estado civil**		
Solteira	18.102	58,61
Casada	5.992	19,40
Viúva	68	0,22
Separada/divorciada	289	0,94
União Estável	6.434	20,83
**Escolaridade da mãe**		
Nenhuma	280	0,9
1 a 3 anos	1.574	5,1
4 a 7 anos	7.888	25,5
8 a 11 anos	16.633	53,8
12 e mais	4.518	14,6
**Tipo de gravidez**		
Única	26.568	74,7
Dupla	8.550	24,0
Tripla ou mais	441	1,2
**Consultas pré-natal**				
Nenhuma	806	0,8
1 a 3	10.672	10,8
4 a 6	36.042	36,6
7 e mais	50.984	51,8
**Semanas gestacionais**				
Menos que 22	342	1,11
22 a 27	1.647	5,37
28 a 31	2.659	8,67
32 a 36	15.281	49,81
37 a 41	10.604	34,56
42 ou mais	146	0,48

*Total de 30.999 BPN, valores abaixo significam que alguns dados não foram preenchidos

De acordo com o mapa de Kernel, os municípios com maior destaque na taxa de BPN foram: regional de Nossa Senhora do Socorro, seguida pela regional de Propriá. As cores indicadas em vermelho e amarelo no mapa representam valores acima da média do estado de Sergipe (Figura 1).

Ao se realizar a análise de séries temporais, por regional de saúde de Sergipe, verifica-se uma tendência decrescente para as regionais de Nossa Senhora da Glória e de Nossa Senhora do Socorro (Tabela 2).

**Figura 1:** Mapa de Kernel da taxa de baixo peso ao nascer (BPN) por município do estado do Sergipe, no período de 2013 a 2023. Aracaju, SE, Brasil, 2024

**Tabela 2:** Análise de série temporal dos casos de baixo peso de recém-nascidos por regiões de saúde de Sergipe, no período de 2013 a 2023. Aracaju, SE, Brasil, 2024

	VPA*	IC^†^		
Região de Saúde	2013-2023	min^‡^	max^§^	tendência
Aracaju	-0,36	-0,02	0,01	estacionária
Estância	-2,24	-0,08	0,04	estacionária
Itabaiana	-0,90	-0,02	0,01	estacionária
Lagarto	-1,07	-0,05	0,03	estacionária
Nossa Senhora da Glória	-3,04	-0,05	-0,01	decrescente
Nossa Senhora do Socorro	-6,82	-0,10	-0,03	decrescente
Propriá	-3,95	-0,09	0,01	estacionária

*VPA = Variação percentual anual; ^†^IC = Intervalo de confiança, ^‡^min = Valor mínimo; ^§^max = Valor máximo

## Discussão

O BPN constitui um importante problema de saúde pública, capaz de gerar consequências graves para a qualidade de vida futura do bebê. Nesse contexto, torna-se fundamental conhecer a realidade local e as possíveis discrepâncias entre diferentes regiões de um mesmo estado, a fim de subsidiar intervenções com medidas eficazes^17^.

O BPN está associado a múltiplos fatores, tais como prematuridade, ausência ou inadequação do pré-natal, idade materna extrema, situação conjugal, baixo nível de escolaridade, paridade, histórico de abortos, ocorrência prévia de filhos com baixo peso e sexo feminino do recém-nascido, além de aspectos relacionados à vulnerabilidade social^18^ ^-^ ^20^.

Neste estudo, a maioria dos recém-nascidos com BPN era pré-termo, do sexo feminino e filho de mãe solteira. Outros autores, ao analisarem o BPN entre recém-nascidos a termo, identificaram associação com sexo masculino, multiparidade e número reduzido de consultas pré-natais, além de pais adolescentes. Também observaram que a maior parte das mães havia concluído o ensino fundamental (64%) e era solteira (68%), achados que corroboram parcialmente os resultados aqui apresentados^21^ ^-^ ^22^. Alguns autores apontam que cor/raça está associada ao BPN quando comparada à raça/cor branca, principalmente em mães economicamente vulneráveis^22^. Esses achados corroboram este estudo.

Autores^23^ observaram associação entre BPN, idade materna, anemia e condições socioeconômicas desfavoráveis. Neste estudo, identificaram-se idades maternas extremas - 10 e 53 anos - associadas ao BPN, reforçando a influência desses fatores.

O mapa de Kernel, ou mapa de calor, é uma técnica amplamente utilizada para visualizar a distribuição espacial de eventos ou taxas, permitindo identificar áreas com maior concentração de um fenômeno - neste caso, o BPN. A aplicação da análise espacial contribui para o planejamento de ações em saúde. A partir do mapa de Kernel, é possível identificar municípios com maior densidade de casos^24^ ^-^ ^25^, o que possibilita a implementação de medidas direcionadas e o aprimoramento do atendimento às famílias dessas regiões^25^.

Neste estudo, a análise por densidade de Kernel permitiu identificar as regiões com maiores taxas de prevalência de BPN, indicando a necessidade de intensificar ações de atenção à saúde da gestante e de educação em saúde. Os municípios em destaque - Rosário do Catete, Telha e Divina Pastora - são de pequeno porte. Segundo dados do Instituto Brasileiro de Geografia e Estatística (IBGE) do ano de 2023, Telha apresenta taxa de mortalidade infantil de 40,82 por mil nascidos vivos e IDH de 0,60; Divina Pastora, IDH de 0,61 e taxa de 32,26; e Rosário do Catete, IDH de 0,63 e taxa de 13,99^13^. Todos apresentam IDH inferior à média estadual (0,70), o que sugere que a vulnerabilidade social influencia as taxas de BPN. Outros autores também destacam que municípios com baixo IDH, especialmente no componente de educação, apresentam piores indicadores de saúde^24^ ^-^ ^26^.

Vale ressaltar que, em 2023, a taxa de mortalidade infantil no Brasil foi de 12,6 óbitos por mil nascidos vivos, enquanto, em Sergipe, esse valor atingiu 18,48 óbitos por mil. Além disso, todos os municípios destacados no mapa apresentam taxas superiores à média nacional, o que evidencia um cenário de maior vulnerabilidade no estado^13^. 

A prevalência de BPN encontrada neste estudo foi de 8,4% no período analisado em Sergipe, valor semelhante ao observado na América Latina (8,7%) e ao registrado no Brasil (8%)^27^. A mortalidade infantil constitui uma das consequências associadas ao BPN e, embora apresente tendência de queda, permanece elevada: segundo o Ministério da Saúde^5^, registra-se uma taxa de 13,3 óbitos por mil nascidos vivos, o que reforça a necessidade de esforços assistenciais contínuos.

Na análise de séries temporais, verificou-se que a maioria das regiões de saúde de Sergipe apresentou tendência estacionária no número de casos de BPN, o que indica ausência de variação significativa ao longo do tempo - o que, nesse caso, não é desejável, pois o ideal seria uma tendência decrescente. 

Mesmo nas regionais que apresentaram municípios em destaque no mapa de Kernel, observou-se tendência estacionária ou, em alguns casos, decrescente, o que evidencia que determinados municípios demandam maior atenção, seja por fatores socioeconômicos, seja por fragilidades nos serviços de saúde.

Apesar de apenas duas regionais apresentarem tendência decrescente, os indicadores de saúde do estado indicam que alguns parâmetros se encontram dentro das metas recomendadas. Exemplo disso é a proporção de gestantes com pelo menos seis consultas de pré-natal realizadas até a 12ª semana gestacional (52%), além da elevada cobertura de Atenção Primária à Saúde (118%, em 2025).

Este estudo apresenta limitações, principalmente relacionadas a possíveis inconsistências no preenchimento do SINASC, incluindo a ausência, em alguns casos, do registro do peso ao nascer. Por se tratar de um estudo ecológico, não foi possível avaliar variáveis individuais importantes, como doenças maternas ou o estado nutricional da gestante, fatores que podem contribuir para a prematuridade.

## Conclusão

Neste estudo, foi possível observar uma tendência estacionária na maioria das regionais de saúde analisadas e em alguns municípios com pontos de maior densidade no mapa de Kernel. Esses locais, provavelmente, requerem maior atenção às ações de saúde para investigação e planejamento em saúde.

## Data Availability

Todos os dados gerados ou analisados durante este estudo estão incluídos neste artigo publicado.

# Translation: Análisis temporal y espacial de los casos de bajo peso al nacer en Sergipe^*^

**DOI:** 10.1590/1518-8345.8113.4869

**Authors:** Vanessa dos Santos Viana, Juliana Schaia Rocha Orsi, Regiane Cristina do Amaral

**Affiliations:** 1 Universidade Federal de Sergipe, Aracaju, SE, Brasil.; 2 Scholarship holder at the Conselho Nacional de Desenvolvimento Científico e Tecnológico (CNPq), Brasil.; 3 Pontifícia Universidade Católica do Paraná, Paraná, PR, Brasil.; 4 Universidade Federal de Sergipe, Departamento de Odontologia, Aracaju, SE, Brasil.

**Keywords:** Recién Nacido Prematuro, Atención Prenatal, Embarazo, Análisis Espacial, Nacimiento Prematuro, Distribución Temporal**.**

## Abstract

**Objetivo:**

realizar un análisis de series temporales y espaciales de los casos de bajo peso al nacer en el estado de Sergipe en el período de 2013 a 2023 para identificar las regiones con mayores tasas.

**Método:**

se utilizaron datos referentes a los nacidos vivos en el estado proporcionados por la Secretaría Estatal de Salud. Se realizaron análisis de mapas de densidad de Kernel por municipio y análisis de series temporales por regiones de salud.

**Resultados:**

en el período analizado se registraron 370.329 nacimientos; de los cuales 30.999 presentaron peso inferior a 2.500 g (8,4%). Según el mapa de Kernel, los municipios con mayor destaque en las tasas de BPN pertenecen a la Regional de Nossa Senhora do Socorro, seguida por la de Propriá. El análisis temporal reveló una tendencia decreciente en las Regiones de Nossa Senhora da Glória y de Nossa Senhora do Socorro.

**Conclusión:**

los casos se presentan en densidades localizadas y con tendencia estacionaria en la mayoría de las Regiones de Salud analizadas.

## Destacados

**(1)** Se identificaron regiones de salud con mayor densidad de casos de BPN. **(2)** La mayoría de las regiones del estado muestran una tendencia estacionaria en las tasas. **(3)** El estado de Sergipe mantuvo una elevada vulnerabilidad social. **(4)** Sergipe presentó un aumento en el porcentaje de casos de BPN (2015, 2018, 2023).

## Introducción

El peso al nacer corresponde al primer pesaje del recién nacido realizado hasta una hora después del parto. De acuerdo con la Organización Mundial de la Salud (OMS)^1^, la proporción de bebés con bajo peso al nacer (BPN) es un importante indicador de salud pública, pues refleja múltiples factores asociados al período gestacional, como la desnutrición materna, las condiciones clínicas y obstétricas, la calidad de la atención prenatal, el nivel de ingresos, la edad materna, las gestaciones múltiples y el estado nutricional.

En 2020, el Fondo de las Naciones Unidas para la Infancia (UNICEF)^2^ estimó que 19,8 millones de recién nacidos -aproximadamente el 14,7% de todos los nacidos vivos en el mundo- presentaron BPN. Estos bebés tienen un mayor riesgo de morir en el primer mes de vida; y, entre los sobrevivientes, se observan consecuencias a lo largo del ciclo vital, como una mayor probabilidad de crecimiento inadecuado, un menor coeficiente intelectual y un mayor riesgo de enfermedades crónicas en la edad adulta, incluidas la obesidad y la diabetes^3^ ^-^ ^4^. Aunque los datos globales de UNICEF indican una tendencia a la reducción del porcentaje de BPN, persisten diferencias regionales significativas. América del Norte y Europa mantuvieron índices estables alrededor del 7,4% en los años 2000, 2010 y 2020, mientras que América del Sur presentó un discreto aumento (8,5%, 8,6% y 8,8%, respectivamente).

En Brasil^5^ ^-^ ^6^, la prevalencia de BPN en 2023 fue de aproximadamente 9,3%. La región Sudeste presentó la mayor tasa (10,1%), seguida por el Centro-Oeste (9,5%), el Sur (9,3%), el Nordeste (8,9%) y el Norte (8,7%). En la región Nordeste, se destacaron Bahía (9,65%), Ceará (9,1%), Piauí y Río Grande do Norte (9,04%), y Sergipe (8,9%). El análisis histórico revela un pequeño aumento en el porcentaje de BPN entre 2015 y 2023: el Nordeste pasó del 7,9% (2015 y 2018) al 8,9% (2023), mientras que Sergipe registró el 8,2%, 8,3% y 8,9% en esos mismos períodos^7^.

La OMS estableció como meta una reducción del 30% en el número de bebés con peso inferior a 2.500 g hasta 2025, lo que equivale a una disminución relativa del 3% anual entre 2012 y 2025, pasando de 20 a cerca de 14 millones de recién nacidos con BPN^8^. Entre las medidas para reducir el BPN en Brasil se recomienda iniciar la atención prenatal en el primer trimestre e incluir, como mínimo, seis consultas: una en el primer trimestre, dos en el segundo y tres en el tercer trimestre^9^. Además, el Ministerio de Salud implementó programas como la Red Cegonha^10^ en el ámbito del Sistema Único de Salud (SUS), con el objetivo de mejorar el acceso, la calidad y la continuidad del cuidado materno-infantil. Sin embargo, a pesar de estos avances, aún se observan disparidades regionales y socioeconómicas en la prevalencia del BPN.

La vulnerabilidad social constituye otro importante determinante del BPN. Según el Centro de Liderazgo Público (CLP)^11^, al evaluar indicadores de salud y de vulnerabilidad, Sergipe ocupa la 22ª posición entre los 27 estados brasileños. Además, el informe “Las Múltiples Dimensiones de la Pobreza en la Infancia y Adolescencia en Brasil”^12^ reveló que el 76,6% de los niños y adolescentes en Sergipe experimentan al menos una dimensión de pobreza y el 22,68% se encuentran en situación de pobreza extrema.

Así, dada la relevancia epidemiológica y social del problema, este estudio tuvo como objetivo analizar la tendencia temporal y la distribución espacial de los casos de BPN en el estado de Sergipe entre 2013 y 2023 para identificar las regiones con las mayores tasas.

## Método

### Local

Sergipe es el estado más pequeño de la Federación, con 182.163 km², está situado en la región Nordeste de Brasil, y tiene una población estimada (2024) de 628.846 habitantes y un Índice de Desarrollo Humano (IDH) de 0,77. La mortalidad infantil en ese estado en 2022 fue de 16,81 muertes por cada mil nacidos vivos^13^.

La Red Materno-Infantil de Sergipe está compuesta por nueve maternidades (cinco públicas y cuatro filantrópicas). Tres están situadas en la capital (Aracaju); y seis en los municipios sedes de cada región de salud (Estância, Itabaiana, Lagarto, Nossa Senhora da Glória, Nossa Senhora do Socorro y Propriá)^14^.

### Definición de la muestra

Se trata de un estudio epidemiológico, retrospectivo, con el uso de datos secundarios de la Secretaría Estadual de Sergipe sobre el número de nacidos vivos en el período de 2013 a 2023. Este estudio fue aprobado por el Comité de Ética en Investigación (CEP), bajo el número CAAE: 77636824.5.0000.5546, dictamen n.º 6.684.748.

Los datos fueron proporcionados por la Secretaría Estadual de Salud de Sergipe, a partir de la base SINASC (Sistema de Información sobre Nacidos Vivos), con información sobre lugar de nacimiento, edad materna, raza materna, estado civil, escolaridad, duración de la gestación, tipo de gestación, tipo de parto, número de consultas, fecha de nacimiento, sexo del bebé, peso del bebé y semana gestacional.

Como criterio de elegibilidad, se incluyeron los registros que contenían el peso del recién nacido. Los siguientes datos fueron considerados: municipio de nacimiento, datos sociales (edad, estado civil, escolaridad, etnia, sexo del bebé, presencia de anomalías), tiempo gestacional, número de gestaciones, tipo de parto (cesárea o vaginal) y número de consultas prenatales.

En el período de 2013 a 2023 se registraron 370.413 nacimientos en el estado de Sergipe; sin embargo, 370.329 informaron el peso al nacer, mientras que los demás aparecían como no rellenados. Del total de nacimientos, 30.999 presentaron peso inferior a 2.500 gramos, es decir, BPN, lo que representa el 8,4% del total de nacidos vivos.

### Tratamiento y análisis de los datos

Se calculó la tasa de BPN, definida como el número de niños con peso inferior a 2.500 gramos (criterio de la OMS) dividido por el número de nacimientos. Esta tasa fue calculada por municipio del estado de Sergipe. Respecto al período gestacional considerado para analizar la asociación con el BPN, el recién nacido se considera prematuro cuando nace con menos de 37 semanas de gestación. Los casos considerados como ignorados o incompletos fueron excluidos.

Se elaboraron mapas de estimación de densidad de Kernel (densidad de puntos en un área geográfica), utilizando un radio de 20.000 metros y un tamaño de píxel de 80, en modo de intervalo continuo, con coordenadas SIRGAS 2000/UTM, zona 24S. Para ello, se utilizó el programa *Quantum Geographic Information System* (QGIS) 3.22.5. Esta estimación permitió identificar los municipios con las mayores tasas de BPN, lo que facilitó visualizar e interpretar los datos geográficos con mayor precisión y eficiencia, apoyando la toma de decisiones y la planificación de acciones.

Asimismo, se realizaron análisis de tendencia temporal, con variación porcentual anual (VPA), con un nivel de significancia del 95%, del número de casos de BPN en las siete regiones de salud de Sergipe (Aracaju, Estância, Itabaiana, Lagarto, Nossa Senhora da Glória, Nossa Senhora del Socorro y Propriá). Para este análisis, se aplicó la regresión de Prais-Winsten, que prevé la corrección de la autocorrelación de primer orden. La variable dependiente fue el logaritmo de los casos, y las variables independientes fueron los años de la serie histórica, utilizando la metodología descrita por Antunes y Waldman^15^. El análisis se realizó en el programa Stata, versión 14.

Las regiones de salud están formadas por municipios fronterizos que comparten identidades culturales, económicas y sociales, redes de comunicación e infraestructura de transportes. La finalidad de estas regiones es integrar la organización, la planificación y la ejecución de acciones y servicios de salud. Las directrices fueron establecidas por la Comisión Intergestora Tripartita (CIT) mediante la Resolución 1 del 29 de septiembre de 2011 y en conformidad con el Decreto 7508 del 28 de junio de 2011 -que organiza el Sistema Único de Salud (SUS), la planificación sanitaria, la atención en salud y la articulación entre el gobierno federal, los estados y los municipios^16^.

## Resultados

Entre los recién nacidos con bajo peso al nacer (BPN), la edad materna media fue de 26 años (±7,4 años), siendo la madre más joven de 10 años y la de mayor edad de 53 años. Con relación al sexo del recién nacido, 16.365 eran del sexo femenino, 14.562 del sexo masculino y 72 estaban registrados como sexo desconocido. Según se observa en la Tabla 1, la mayoría eran de raza parda, solteras, con 8 a 11 años de escolaridad y 32 a 36 semanas de gestación.

**Tabla 1:** Variables sociodemográficas del total* de bajo peso al nacer (BPN) en el estado de Sergipe, 2013-2023. Aracaju, SE, Brasil, 2024

Edad materna	N*	%
10 a 17 años	3.856	12,4
18 a 35 anos	23.025	74,3
más de 35 años	4.118	13,3
**Raza**		
Blanca	2.923	9,58
Negra	1.600	5,24
Amarilla	265	0,87
Parda	25.663	84,12
Indígena	55	0,18
**Estado civil**		
Soltera	18.102	58,61
Casada	5.992	19,40
Viuda	68	0,22
Separada/divorciada	289	0,94
Unión estable	6.434	20,83
**Escolaridad materna**		
Ninguna	280	0,9
1 a 3 años	1.574	5,1
4 a 7 años	7.888	25,5
8 a 11 años	16.633	53,8
12 o más	4.518	14,6
**Tipo de gestación**		
Única	26.568	74,7
Doble	8.550	24,0
Triple o más	441	1,2
**Consultas prenatales**		
Ninguna	806	0,8
1 a 3	10.672	10,8
4 a 6	36.042	36,6
7 o más	50.984	51,8
**Semanas gestacionales**		
menos de 22	342	1,11
22 a 27	1.647	5,37
28 a 31	2.659	8,67
32 a 36	15.281	49,81
37 a 41	10.604	34,56
42 o más	146	0,48

*Total de 30.999 BPN; valores inferiores indican datos incompletos

De acuerdo con el mapa de Kernel, los municipios con mayor destaque en la tasa de BPN fueron la Regional de Nossa Senhora do Socorro, seguida de la Regional de Propriá. Los colores señalados en rojo y amarillo en el mapa representan valores superiores al promedio del estado de Sergipe (Figura 1). Al realizar el análisis de series temporales por regional de salud de Sergipe, se observa una tendencia decreciente en las regionales de Nossa Senhora da Glória y de Nossa Senhora do Socorro (Tabla 2).

**Figura 1:** Mapa de Kernel de la tasa de bajo peso al nacer (BPN) por municipio del estado de Sergipe, en el período de 2013 a 2023. Aracaju, SE, Brasil, 2024

**Tabla 2:** Análisis de serie temporal de los casos de bajo peso al nacer por regiones de salud de Sergipe, en el período de 2013 a 2023. Aracaju, SE, Brasil, 2024

Región de Salud	VPA*	IC^†^ min^‡^	IC^†^ máx^§^	Tendencia
Aracaju	-0,36	-0,02	0,01	estacionaria
Estância	-2,24	-0,08	0,04	estacionaria
Itabaiana	-0,90	-0,02	0,01	estacionaria
Lagarto	-1,07	-0,05	0,03	estacionaria
Nossa Senhora da Glória	-3,04	-0,05	-0,01	decreciente
Nossa Senhora do Socorro	-6,82	-0,10	-0,03	decreciente
Propriá	-3,95	-0,09	0,01	estacionaria

*VPA = Variación porcentual anual; ^†^IC = Intervalo de confianza; ^‡^min = Valor mínimo; ^§^máx = Valor máximo

## Discusión

El bajo peso al nacer (BPN) constituye un importante problema de salud pública, capaz de generar consecuencias graves para la calidad de vida futura del recién nacido. En este contexto, resulta fundamental conocer la realidad local y las posibles discrepancias entre regiones de un mismo estado, con el fin de subsidiar intervenciones basadas en medidas eficaces^17^.

El BPN está asociado a múltiples factores, tales como prematuridad, ausencia o inadecuación de la atención prenatal, edad materna extrema, situación conyugal, bajo nivel de escolaridad, paridad, antecedentes de abortos, historia previa de hijos con bajo peso, sexo femenino del recién nacido, además de aspectos relacionados con la vulnerabilidad social^18^ ^-^ ^20^.

En este estudio, la mayoría de los recién nacidos con BPN eran pretérminos, del sexo femenino y de madres solteras. Otros autores, al analizar el BPN entre recién nacidos a término, identificaron asociación con el sexo masculino, la multiparidad y un número reducido de consultas prenatales, además de la paternidad en la adolescencia. También observaron que la mayoría de las madres había concluido la educación primaria (64%) y que era soltera (68%), hallazgos que corroboran parcialmente los resultados aquí presentados^21^ ^-^ ^22^. Algunos autores señalan que la raza/color está asociada al BPN cuando se compara con la raza blanca, especialmente entre madres económicamente vulnerables^22^. Estos hallazgos corroboran los encontrados en este estudio.

Otros autores^23^ observaron una asociación entre BPN, la edad materna, la anemia y las condiciones socioeconómicas desfavorables. En este estudio se identificaron edades maternas extremas -10 y 53 años- asociadas al BPN, lo que refuerza la influencia de estos factores.

El mapa de Kernel, o mapa de calor, es una técnica ampliamente utilizada para visualizar la distribución espacial de eventos o tasas, lo que permite identificar áreas con mayor concentración de un fenómeno -en este caso, el BPN. La aplicación del análisis espacial contribuye a la planificación de acciones en salud. A partir del mapa de Kernel, es posible identificar municipios con mayor densidad de casos^24^ ^-^ ^25^, lo que permite implementar medidas dirigidas y mejorar la atención a las familias de estas regiones^25^.

En este estudio, el análisis por densidad de Kernel permitió identificar las regiones con mayores tasas de prevalencia de BPN, lo que indica la necesidad de intensificar acciones de atención a la salud de la gestante y de educación en salud. Los municipios en destaque -Rosário do Catete, Telha y Divina Pastora- son de pequeño porte. Según datos del Instituto Brasileño de Geografía y Estadística (IBGE) de 2023, Telha presenta una tasa de mortalidad infantil de 40,82 por mil nacidos vivos y un IDH de 0,60; Divina Pastora, un IDH de 0,61 y una tasa de mortalidad infantil de 32,26; y Rosário do Catete, un IDH de 0,63 y una tasa de mortalidad infantil de 13,99^13^. Todos tienen un IDH inferior al promedio estatal (0,70), lo que sugiere que la vulnerabilidad social influye en las tasas de BPN. Otros autores también destacan que los municipios con IDH bajo, especialmente en el ítem escolaridad, presentan indicadores de salud más desfavorables^24^ ^-^ ^26^.

Cabe destacar que en 2023 la tasa de mortalidad infantil en Brasil fue de 12,6 muertes por mil nacidos vivos, mientras que la de Sergipe fue de 18,48 muertes por mil. Además, todos los municipios destacados en el mapa presentan tasas superiores al promedio nacional, lo que evidencia un escenario de mayor vulnerabilidad en el estado^13^.

La prevalencia de BPN encontrada en este estudio fue del 8,4% en el período analizado en Sergipe, valor similar al observado en América Latina (8,7%) y al registrado en Brasil (8%)^27^. La mortalidad infantil constituye una de las consecuencias asociadas al BPN y, aunque presenta tendencia decreciente, continúa elevada. Según el Ministerio de Salud^5^, se registra una tasa de 13,3 muertes por mil nacidos vivos, lo que refuerza la necesidad de esfuerzos asistenciales continuos.

En el análisis de series temporales, se verificó que la mayoría de las regiones de salud de Sergipe tuvo tendencia estacionaria en el número de casos de BPN. La tendencia estacionaria indica la ausencia de variación significativa a lo largo del tiempo -lo cual, en este caso, no es deseable, pues lo ideal sería una tendencia decreciente.

Incluso en las regionales que presentaron municipios destacados en el mapa de Kernel, se observó una tendencia estacionaria o, en algunos casos, decreciente, lo que evidencia que determinados municipios requieren mayor atención, ya sea por factores socioeconómicos o por fragilidades en los servicios de salud.

A pesar de que solo dos regionales mostraron una tendencia decreciente, los indicadores de salud del estado evidencian que algunos parámetros se encuentran dentro de las metas recomendadas. Ejemplo de ello es la proporción de gestantes con al menos seis consultas prenatales realizadas hasta la 12ª semana gestacional (52%), así como la elevada cobertura de Atención Primaria en Salud (118% en 2025).

Este estudio presenta limitaciones, principalmente relacionadas con posibles inconsistencias en el llenado del Sistema de Información sobre Nacidos Vivos, incluida la ausencia, en algunos casos, del registro del peso al nacer. Por tratarse de un estudio ecológico, no fue posible evaluar variables individuales importantes, como las enfermedades maternas o el estado nutricional de la gestante, factores que pueden contribuir a la prematuridad.

## Conclusión

En este estudio fue posible observar una tendencia estacionaria en la mayoría de las regiones de salud analizadas y en algunos municipios con puntos de mayor densidad en el mapa de Kernel; estos probablemente requieren mayor atención en las acciones de salud para la investigación y la planificación sanitaria.

## Data Availability

Todos los datos generados o analizados durante este estudio están incluidos en este artículo publicado.
